# Do Playful Parenting Programs Implemented at Scale Improve Caregiver Practices and Child Development?

**DOI:** 10.3390/children12091241

**Published:** 2025-09-16

**Authors:** Carina Omoeva, Rafael Contreras Gomez, Rachel Hatch, Frances Aboud, Ania Chaluda, Given Hapunda, Karma Choden, Francis Sichimba, Ksenija Krstić, Jill Popp

**Affiliations:** 1 FHI 360 Research and Evaluation, Washington, DC 20036, USA; rcontrerasgomez@fhi360.org (R.C.G.); rhatch@fhi360.org (R.H.);; 2 Department of Psychology, McGill University, Montreal, QC H3A 1G1, Canada; frances.aboud@mcgill.ca; 3 Department of Psychology, University of Zambia, Lusaka P.O. Box 32379, Zambia; 4 Independent Consultant, Thimphu 11001, Bhutan; 5 Department of Psychology, University of Belgrade, 11000 Belgrade, Serbia; 6 LEGO Foundation, DK-83, 7190 Billund, Denmark

**Keywords:** parenting, early childhood development, program evaluation, scaling up, Serbia, Zambia, Bhutan

## Abstract

**Background/Objectives**: As an independent research group, we examined parent and child outcomes of three different parenting programs delivered at scale. The programs were implemented in Bhutan, Serbia and Zambia by different organizations. **Methods**: Mixed methods included a caregiver interview using the HOME Inventory, a direct child assessment using the Global Scales of Early Development (GSED) and focus group discussions with caregivers (FGD). Sampled mothers and children were randomly selected for the HOME/GSED: Bhutan *n* = 432, Serbia *n* = 636, Zambia *n* = 1024. Over 40 mothers and fathers of children under 3 years were purposively selected for FGD. Intention-to-treat and secondary regression analyses of attendees and non-attendees were conducted on the HOME and GSED; FGDs were subject to content analysis. **Results**: Parenting practices were found to be minimally (Bhutan) or modestly (Zambia) higher for caregivers who attended group sessions. Caregivers in Serbia who recalled receiving play messages had higher HOME scores. Child outcomes showed small (Bhutan) or no differences (Serbia, Zambia) associated with participation. **Conclusions**: Explanations focused on limits to program participation in scaled programs, the need for pilot evaluations to ensure that the program design is effective, and the need to monitor delivery quality and other implementation processes.

## 1. Introduction

Approximately 43% of children under 5 years in low- and middle-income countries do not attain mental development milestones as expected [1], and there is consensus that this is the result of inadequate levels of nutrition and psychosocial stimulation [2]. To promote parental practices that help to remediate child outcomes, organizations and governments have implemented parenting programs for caregivers of children under 3 years. Parenting programs can be defined as “a set of activities or services directed at parents/caregivers, with the objective of improving parent–child interactions and the overall quality of parenting that a child receives” [3], in this case to improve the child’s mental and motor development. Many different programs exist that overall have yielded moderate size effects according to meta-analyses [4]. Most of the interventions currently published and reported in meta-analyses have been evaluated as randomized controlled trials with relatively small sample sizes. Only a few have transitioned to scale with the intention of transferring the program to the government and expanding geographically [5].

The objective of this study was to examine caregiver and child outcomes of three parenting programs in Bhutan, Serbia and Zambia that transitioned to scale over the course of four years. The study was conducted by an independent research and learning group (FHI 360) using mixed methods. Each of the country organizations adapted, implemented and scaled a program that they expected to be effective in their setting. Because programs and country settings were different, there was no intention to compare them, but rather to determine the effectiveness of each at scale. While the implementation process in each country has been studied and published [6,7], it is also important to know whether programs were effective in improving parent and child outcomes.

### 1.1. Background on Outcomes

#### 1.1.1. Parenting Practices

Parenting practices for programs aimed at improving children’s mental development refer mainly to those directed at responsive stimulation. Responsive stimulation refers to activities and materials that arouse the child’s sensory system and are contingent on the child’s cues of interest and ability [4]. Theoretical explanations for the impact of responsive stimulation on children’s mental development rely on the arousal of networks in the brain elicited by adult–child interactions [1]. The most commonly used measures of parenting practices related to child development assess the provision of stimulating activities, responsive interactions and play materials. This includes predominantly the HOME Inventory-Infant and Toddler version (Home observation of measurement of the environment) [8] which is strongly correlated with a child’s mental development.

Two meta-analyses of published interventions using the HOME to evaluate changes in psychosocial stimulation parenting practices found effect sizes of d = 0.57 and d = 0.33 across 15 and 40 studies, respectively [4,9]. Generally, improvements were stronger in LMIC compared to high-income countries [4]. It is noteworthy that improvements in parental practices, knowledge and intervention message recall are frequent mediators of changes in child developmental outcomes [4]. This suggests that interventions focusing on improving parental stimulation practices impact children’s mental development by way of actual changes to stimulation practices.

#### 1.1.2. Child Developmental Outcomes

The effect of a parenting program is largely evaluated in terms of its impact on the child, especially the child’s cognitive and language development. Changes in parental practices are not always sufficient to improve the child’s development. To date, child outcomes have been assessed with the Bayley Scales of Infant and Toddler Development [10], and, more recently, with the Global Scales of Early Development (GSED) [11]. The GSED includes subscales for cognitive, language and motor development and is the ascendant measure going forward because materials can be sourced locally. Still, as a direct assessment of the child, it requires systematic training of assessors.

Meta-analyses demonstrate that parenting programs in LMIC have a moderate effect size on children’s mental development—stronger than effect sizes in HIC. The effect sizes are d = 0.41 for cognitive development, 0.35 for language development, 0.26 for motor development, and 0.24 for social-emotional development [4]. Motor development is weaker than the others, as is social–emotional development for which measures were caregiver reports and therefore less valid. Although a moderate effect size is graded as high by WHO guidelines [12], approximately 40% of the studies did not yield even a small effect size greater than 0.20 for cognitive and language development. Consequently, substantial effects on child outcomes are not guaranteed without a strong program.

The theory linking parental practices and child development has been elaborated through pathways by which responsive stimulation enhances development [13,14]. Programs that promote responsive stimulation typically include play materials that parents are encouraged to provide from home and child-directed talk used by adults when interacting with children. Play materials intrinsically arouse curiosity and exploration, which in turn influence mental and motor development. They do this directly, for example, as the child learns to solve the problem of stacking blocks without letting them fall, or indirectly by activating the link between the fine motor and the cognitive parts of the brain. Child-directed talk used by adults when conversing with children similarly expands the child’s language and socio-emotional development. This form of responsive talk expands language directly, for example, by linking verbal phrases to the child’s actions, and indirectly by activating the language parts of the brain or activating the language and the cognitive or motor parts of the brain. Consequently, parenting programs that show parents how to use colorful moving play materials and verbal communication in a responsive manner elicit in their child exploration, reciprocal communication, and brain growth. Programs have a greater impact if they encourage parents to do activities daily with their child and encourage them to provide novel playthings and new words or songs. The practical side of these theoretical pathways is revealed in two ways: One is that many parents and providers are unaware of the importance of providing responsive stimulation [15] and curricula do not include behavior change techniques such as demonstrations and coaching to teach and maintain responsive stimulation [16].

#### 1.1.3. Program Implementation 

We now understand some potentially critical ingredients of a strong parenting program. It requires intervention features suitable for the audience, features that need to be assessed ahead in a situation analysis [15]. For example, specific features are needed for an audience of caregivers who are not aware of brain development and the importance of play and communication. They include a curriculum with sufficient focus on responsive stimulation, adequate dosage of contact time with parents, and behavior change techniques such as demonstrations, along with parental practice and coaching with the child. In a recent review of parenting interventions [16], contact time varied considerably: when the modality was either home visits or group sessions, caregivers in LMIC were given a total of over 40 h contact time; mixed home and group sessions offered 22 h and clinic visits averaged seven hours. Programs with activities that included three or more behavior change techniques to modify parenting practices had stronger effects than those using less than three. Because only 50 interventions were included in this review, its conclusions about associations with child outcomes and parenting practices are presented with caution.

Other implementation processes may be importantly related to parent and child outcomes, but associations are less clear. For example, concerning the workforce, training may need to be lengthier and supervision more extensive when the providers are volunteers compared to paraprofessionals or professionals. However, in the review, most providers were trained for 7 days or less and supervised less than weekly [16]. This may be inadequate if the providers, like caregivers, have little expertise or experience in early childhood matters [15]. Guidance for inexperienced providers is often available in the form of a manual that outlines specific activities to engage parents and their children—activities that incorporate behavior change techniques. Finally, monitoring quality delivery is important to maintain accountability and to ensure that providers receive feedback on their performance [6,16].

#### 1.1.4. Evaluations at Scale

A review of published parenting programs in LMIC that are either in-transition to scale or actually scaled found that most were universal rather than targeted, and conducted in Latin America or Asia, with none in Africa [5]. Seven out of 13 had completed an evaluation of the scaled program. For example, programs in Peru and Chile had positive outcomes for parents and children though small effect sizes of 0.10 [17,18], whereas those in Brazil were not effective [19,20,21]. The Learning Clubs program in Vietnam had positive effects on children but did not reduce depression in mothers [22]. All these scaling programs used a workforce that was integrated somehow with the government, though some were engaged at the national government level [18] while others were engaged with municipal or district governments [20,21]. Most noted that reach and dosage was less than intended because many eligible families did not participate or participated less than intended. Regardless of the effect of the intervention, researchers felt that evaluations were important to conduct and expected that the program would be revised accordingly. While success of scaling a program might be judged in terms of adoption by the government, sustained over time, and workforce integration into the health system, an important goal was to improve the practices of parents and the development of their children.

The situation regarding experience with parenting programs is somewhat similar in each of our three countries, in that small pilot or effectiveness programs had been evaluated but not larger scaled programs. They were selected by the donor from a large number of grant applicants because they showed promise in reaching scale with an effective program. Programs were intended to promote “playful parenting” namely parental provision of stimulation to children under 3 years of age, in particular, sensory and motor stimulation that engages the child actively and socially yet is enjoyable and developmentally beneficial. The government of Bhutan had previously experimented with parenting and preschool programs delivered by UNICEF. Though not scaled, participation in both programs was found to enhance home learning activities and child outcomes [23]. Save the Children improved on the parenting program and worked closely to gain buy-in from the government for their enhanced program. In Serbia, UNICEF had been working previously with the government to implement a parenting program but its evaluation was neither rigorous nor positive [24]. Zambia implemented UNICEF’s Care for Child Development program which in the past was found to be only partially effective in only some countries [25]. Several small RCTs delivered in the Eastern Province yielded positive parent and small child outcomes in Zambia though they were not scaled [26].

Thus, there is a lack of information on how parents and children benefit from parenting programs in these and other sites as they operate at scale. Although there is considerable evidence from small controlled studies that parenting practices and children’s development can improve as a result of an intervention, in many cases they do not. Scaling poses additional challenges that may reduce impacts. The challenges associated with scaling are not well known though they likely concern implementation processes to expand geographically and to transfer the program to the government. There may also be a mismatch between the needs and demands of the community and the program design. Although implementation processes require their own in-depth evaluation, some challenges such as attendance are often examined in outcome studies. The current study examined effects of three different parenting programs operating at scale. Each was evaluated on its own, as their goal was to provide an effective program at scale.

### 1.2. Research Questions and Hypothesis

The following research questions were posed:

1. What were the prevailing parenting beliefs around play, communication and responsive stimulation among the target population before the programs started?
2. What difference in parenting practices resulted from the program being implemented at scale?
3. What differences in children’s development resulted from the program being implemented at scale?

We hypothesized that caregivers who participated in the parenting program would demonstrate improved parenting practices, and that their children would show higher developmental scores, compared to those who did not participate.

## 2. Materials and Methods

The study employed mixed methods to examine caregiver and child outcomes for three Playful Parenting programs implemented in three countries between 2020 and 2024. Qualitative data were collected at baseline in order to explore how parents prioritized responsive stimulation in the form of play and communication with young children. Qualitative data were collected at endline to explore what parents had learned from the program. Quantitative methods were used to evaluate parenting practices and children’s development at scale (endline).

Regression analysis was performed to estimate the relationship between participation in the program implemented at scale with caregiver and child outcomes. Sensitivity checks examining possible bias in participation are included.

### 2.1. Study Setting

The study took place between 2020 and 2024 in three countries, Bhutan, Serbia, and Zambia. The three countries differ substantially in their geography and development context: Serbia has a human development index (HDI) of 0.805 ranked 65 out of 193 countries; Bhutan’s HDI score is 0.698 ranking 125 out of 193; Zambia’s HDI is 0.569 ranking 153. Given that the HDI is based on life expectancy at birth, literacy, and Gross National Income per capita, the curriculum and dosage of Playful Parenting programs necessarily had to be adjusted to the context in order to be effective.

All three programs received grants that sought to help them take to scale their interventions focused on play and responsive stimulation for caregivers with children 0–3 years. Goals for geographic scale varied across the country contexts, ranging from two districts in Zambia to 34 municipalities in Serbia, to nationwide implementation in Bhutan. Goals for the number of families to be reached also differed, with over 16,000 in Bhutan and 50,000 in both Serbia and Zambia. The programs varied in their structure, delivery agents, intensity and frequency of contact with caregivers, and the amount of content on play and responsive stimulation in their curriculum and training materials (see Table 1).

#### 2.1.1. Bhutan Setting

In Bhutan, the Ministry of Health has the primary responsibility for services to newborns through the first 1000 days, which it accomplishes through a network of primary care centers (PHC) and outreach centers (ORC) across its 20 districts. The PHCs and ORC were staffed by health assistants (HAs), who are trained health professionals. HAs were responsible for routine care at PHC’s where they were sometimes the sole professional health provider, and for monthly outreach days at the ORC’s, which required them to travel to remote locations within their district or catchment area.

In Bhutan, the Prescription to Play (P2P) program generally followed the Save the Children Building Brains curriculum [27]. HAs received 12-day training and were given detailed manuals with session plans for 12 monthly group sessions for caregivers within their catchment area. Caregivers with children 0–36 months were invited to attend the group sessions that followed their growth monitoring. Group sizes varied from 5 to over 40 caregivers and averaged around 9–15. Play was the explicit focus in 9 out of 12 monthly sessions. Following a government priority, the program also included a component for screening for developmental delays, followed by individualized counseling for caregivers whose children were flagged in the screening, as well as those who had missed the monthly group sessions.

Observations of provider training, and subsequently, of group sessions with caregivers showed that the quality of sessions, as measured by the HA’s coverage of content, demonstration and coaching of caregivers, improved over time when weaknesses were identified in the first year [6]. In in-depth interviews with the research team, HAs indicated that while motivated and enthusiastic about the program goals, they often struggled with the additional workload and outreach responsibilities associated with enrolling caregivers into sessions and tracking attendance. During the program implementation period, some HA’s received monitoring visits from the program team; however, no institutionalized coaching and supervision was available specifically on the parenting session implementation.

#### 2.1.2. Serbia Setting

In Serbia the responsibility for early childhood care at the national level was split between the Ministry of Health and Ministry of Education; however, much of the implementation and administrative decision making was cascaded down to the level of its 145 municipalities. The Playful Parenting program was implemented by UNICEF in 34 municipalities selected through an application process. The Standing Conference of Towns and Municipalities was a key counterpart supporting the rollout of the program to the selected areas, working with local authorities to identify the health providers for training on Playful Parenting. The program took advantage of an existing institutional practice of home visits to families of newborns by nurses from a local primary healthcare facility. The home visiting nurses (HVN) were already mandated by the public health system to visit families five times in the first 15 days of the child’s life, followed by two visits in the first two years of their life. The Playful Parenting program provided the HVNs with additional six-day training on the concepts of early childhood development, play and responsive stimulation, and encouraged them to integrate the messages on play into their home visits to families with newborns and infants.

The decentralized nature of implementation in Serbia left a lot of variability at the municipal level for how the program was rolled out. Notably, across all municipalities, HVNs were not provided with job aids, manuals, or performance expectations on how the play content was to be integrated in a regular home visit. Master trainers were available to provide coaching and support to some of the HVN providers, but their coverage was limited.

#### 2.1.3. Zambia Setting

In Zambia, UNICEF implemented the Care for Child’s Healthy Growth and Development curriculum [28], drawing on an existing cadre of Community-Based Volunteers (CBVs) under the auspices of the Ministry of Health. The UNICEF program sought to scale up within two districts of the Eastern Province, selected in consultation with the Ministry of Health based on estimated need for services. ECD had been recognized as a longstanding priority of the Government of Zambia; the UNICEF program innovation was the integration of play and early responsive stimulation messaging into the broader framework of care.

Under CCD, the would-be frontline providers were recruited among the CBVs in the two target districts and trained to carry out seven visits to families with children ages 0–24 months. Initial training lasted 5 days, during which the CBVs were instructed in carrying out home visits to families in their catchment area, and provided with a set of counseling cards with play and communication content along with cards on health and nutrition. Each counseling card included one or two illustrated play and communication activities, as well as prompts for caregivers to talk about how they play and talk with their child. In the final year the program added a group mode of delivery; group sessions were offered mainly at health facilities during monthly growth monitoring and immunization days.

The CBVs received no remuneration from the program, save for limited transportation allowance for training days and days of scheduled supervisory check-in. A CBV policy was in development during the period of the study. Supervision was administrative in nature; no coaching or quality monitoring was provided. Observations conducted by the research team in later stages of program implementation showed that CBVs had limited understanding of core aspects of responsive stimulation, and needed a greater degree of supportive supervision [7].

### 2.2. Design and Participants

To be eligible for participation, households must have had a child aged 6–24 months during at-scale data collection. Treatment sites were non-randomly assigned by the implementing partner along with the government; we selected comparison sites that in some cases were waitlisted to receive the intervention and demographically similar. Sample size estimation aimed for an effect size of 0.30, power 0.80. Eligible children in each intervention site and comparison sites were randomly selected from a list of households. Although it was expected that caregivers of eligible children in intervention sites would have participated in the program in the previous 12 months, some reported no participation. Program coverage data will be presented in Results.

In Bhutan, scale up proceeded in two phases and encompassed the entire country; a sample of 432 households was drawn from seven districts: three from Phase 1 of national rollout of the program (Tsirang, Bumthang, and Mongar) and four from Phase 2 (Dagana, Paro, Pema Gatshel, and Samtse). At scale the sample included families from these districts who had participated in the program over the prior year (intervention) and families who reported that they had not participated (comparison).

In Serbia, the program was scaled up to 34 out of 145 municipalities through a selection process using a series of merit criteria; the study sample of 636 families with children under 18 months of age was drawn from eight municipalities that were selected for the program (Cuprija, Mionica, Prijepolje, Svilanjnac, Titel, Vladimirici, Zabali, and Zemun) and four that were comparison sites just below the passing score (Kucevo, Pecinci, Smederevo, and Uzice). Thus, at scale those in the eight municipalities served as intervention and those in the four served as comparisons.

In Zambia, the program was scaling up within two districts in the Eastern province (Katete and Petauke); two additional districts were waitlisted to receive the program (Chipata and Chongwe). The Zambia sample was drawn from all four districts, two program-receiving and two comparisons.

Samples of mothers and fathers in intended sites of the three countries were purposively selected for focus groups discussions (FGD). Over 40 parents of children under 3 years of age from each country participated.

### 2.3. Measures and Procedures

#### 2.3.1. Focus Group Discussions with Caregivers from Communities

Focus group discussions (FGD) were organized with purposively selected caregivers in each country to examine their beliefs around parenting and play prior to scale up (baseline for the purpose of this study) and what they had learned from the programs when the program operated at scale (endline). The discussions were separated by sex, with equal numbers of female and male caregiver groups organized. The baseline groups included parents targeted by the respective parenting programs, although they may not have eventually participated. The FGDs lasted 60–120 min and covered questions on what children needed for development and parenting resources available to the caregivers. The discussions were moderated by one trained person from a local data collection firm, and notes were taken by a second researcher. They were audio recorded and consent was obtained from all participants. Transcripts were then translated from local languages in each country into English and subsequently coded and analyzed.

Endline groups included purposively selected male and female caregivers who may or may not have participated in the program. Male caregivers, in particular, were less likely to have been reached by the programs though their partners may have. Those who participated were asked what they had learned from the program.

#### 2.3.2. Caregiver Practices: HOME Inventory

To measure parenting practices, in Bhutan and Zambia, we used the Home Observation Measurement of the Environment, a 45-item inventory covering the quality and quantity of psychosocial stimulation provided by the child’s home life [8]. It is the most commonly used measure of parenting practices for children’s development and the subject of several meta-analyses [4]. The HOME Inventory includes an interview with the caregiver on stimulating activities, an observation of playthings, and an observation of the caregiver and child interaction during the same visit. In Serbia, we administered the HOME inventory at endline, and a different instrument before scale-up based on caregiver report only because of COVID restrictions. The instrument used in Serbia incorporates the main stimulation items from the HOME with 15 items on playthings and stimulating activities [29]. The items for all measures were translated into the local language. Responses for the stimulation measures (HOME; Early Learning Opportunities) were Yes (1) or No (0). An interview with the primary caregiver in each site included demographic questions concerning parents’ age, education, and assets, and the child’s age and gender. Interviewers were trained and practiced using locally relevant examples. The entire interview and observation were conducted at the child’s home.

#### 2.3.3. Child Development: Global Scales for Early Development

At endline, we assessed children’s mental and motor development using the Global Scales for Early Development—Long form (GSED), a measure launched by the WHO in 2023 [11]. The GSED is a direct assessment of children’s cognitive, language, and motor development using age-specific tasks, which is then scored on the number of items they correctly complete. The items are ordered by difficulty; children begin at an age-specified item and finish when they are unable to master four items in a row. As children age, they are expected to complete more items in developmental assessments, resulting in higher total or raw scores with increasing age (these are referred as d-scores in the GSED). D-scores can be standardized into Development-for-Age z-scores (DAZ) to allow for comparison between samples from different ages. Like measures such as height-for-age, the DAZ is calculated relative to a reference population and is normally distributed with a mean of 0. In other words, for each child we ask: how many standard deviations (SD) is a child’s d-score away from the mean d-score among children in their age group in a reference population? Within a country context, we are able to compare the DAZ for the children of families that are targeted by and receiving the interventions, to outcomes of children whose families have not been enrolled in parenting programs. The GSED was found to correlate significantly with the CREDI in Bhutan and with the ASQI in Serbia.

Training of assessors took place at sites in their country over the course of one week. Four Bhutanese assessors, four Serbian assessors and eight Zambian assessors were trained by an international researcher who was previously trained by WHO. Materials for the tasks were purchased or made locally. Although the training was conducted in English, in which all trainees were proficient, words spoken to the child were subjected to a process of translation, back-translation, and correction with the local language. Videos and zoom meetings of the trainees’ administration of the GSED allowed WHO trainers to verify their competence. Practice on the final day was conducted with children between 6 and 36 months of age. Inter-rater reliabilities were calculated at over 80% using administrator-observer pairs. The test was conducted at the child’s home with the caregiver sitting close by.

### 2.4. Methods of Analysis

Focus Group Discussion transcripts were subjected to a detailed content analysis using the following process. A lead investigator created a set of codes pertaining to each question, coders were trained how to apply the codes and then coded a set of ten excerpts from one transcript. Their percentage agreement with the lead investigator ranged from 71% to 79%. This was considered acceptable and so they moved to coding on Dedoose 9.0.15 software. Each meaningful phrase was coded. To reduce the number of codes, when coding was complete, codes with low frequencies were combined into higher-order codes and into codes that were conceptually valuable to the research question. For each code, we recorded frequencies of comments that participants mentioned in answer to the moderators’ questions. Representative quotes from participants were noted.

Quantitative data from the HOME Inventory and GSED were analyzed as follows: a series of regression analyses was applied to our endline samples in Bhutan, Serbia and Zambia. The regressions are ordinary least squares models: in the first set, the dependent variable is caregiver practices measured through the total HOME score; in the second set, the dependent variable is child outcomes measured through the GSED for age z-score (DAZ), standardized to the international reference group [11]. The regression models first examined intention-to-treat differences, namely due to residing in program sites or comparison sites (Serbia and Zambia). Because participation in the program was less than intended, subsequent regression analyses compared those who reported receiving the program with those who were eligible but reported not receiving the program, within the target districts. Bhutan had a nation-wide scale-up so there was no comparison group at endline; consequently, only the second regression analysis based on participation was conducted. Background demographic characteristics, including caregiver gender, age, education and socioeconomic status, as well as child gender and age are included as control variables; district fixed effects are included for models examining program participation within target areas. Effect sizes were calculated using the Campbell Collaboration calculator of d for regression analyses.

Sensitivity checks examine possible selection bias in program participation, by comparing those who participated and those who did not on a series of demographic variables.

## 3. Results

Results are presented to address the three research questions. First, analyses of FGD at before scale-up revealed parents’ beliefs about child development and resources available to support them. This set the context for sites before programs began. Then, we present the demographics of baseline and endline samples of caregivers and children who provided outcome data. Then, for each country separately, data from regression analyses on parenting practices and child development at scale-up are presented. To supplement these findings, we present data on caregivers’ views on what they learned.

### 3.1. Caregivers’ Beliefs Before Scale-Up

Analysis of FGDs across two of the three countries—Bhutan and Zambia—found that caregivers initially understood the question of growth and development in terms of physical growth (see Table 2). So, commonly they mentioned provisions such as food, vaccination and hygiene. When encouraged to talk more on mental development like thinking and language, they talked about providing play, playthings, and talking with the child. Caregivers in Bhutan also uniquely emphasized that providing children a social life with peers helped their social and language development. In Serbia, they jumped right from the start with comments about play, talking, love, and generally giving attention.

When asked what types of support and resources were needed by the parents of young children, comments varied by country, as Table 3 shows. In Bhutan, participants noted that they needed caregiver training and/or counselling along with trained people to look after their children (e.g., childcare workers, babysitters). “We need trained people who can guide and care for children. We need children’s toys so that caregivers can relax while the child is playing. What do we have? Only a park for children and nothing else” [Haa mothers]. “We need a day care where support for mental development can be provided. Most important is if there is a playground. When more children gather, their mental development is also fast” [Punakha mothers]. Parents in Serbia similarly noted the need for training of caregivers, and less so the need for resources. By contrast, in Zambia, parents highlighted the need for material resources, including food, followed by playthings in the home.

Regarding what resources were currently available to them and used by them, only a few caregivers in Bhutan and Zambia provided answers. Overall most FGD participants noted the health system. Only in Serbia were information apps also available on the internet and used. When asked if they knew about organizations offering such services to parents, few participating caregivers knew of the parenting programs that were starting up in their communities. Only one caregiver in Bhutan had heard of the parenting program offered by Save the Children and this is what she described: “I heard you sing songs in front of small children, spread vegetables for them to recognize, let them play with knives, plates and mugs and let the children do what they want. I think such activities will speed up children’s learning process” [Punakha mother]. All participants in Bhutan expressed a need for parental training in childcare, but demand for such services was infrequently addressed. The nature of such services was not clearly articulated except to state that training and counselling in childcare would be useful. Similarly, in Zambia those from Petauke district were aware of the UNICEF Care for Healthy Growth and Development program but knew little about it. They were looking forward to participating in the parenting program and felt there was a need.

Serbian caregivers knew about home visits by community nurses, noted mainly for the help they offered regarding the physical care of the newborn, and less so about the components addressing responsive play. They were eager to meet with other parents in small groups where a professional might facilitate discussions: “Maybe parents can talk to each other about it, with an expert there. At least once a month, or to have talks organized and everyday socializing. To share experience” [Bor mother].

In summary, caregivers in Zambia and Bhutan prioritized the provision of food and gave some but less emphasis to responsive stimulation in the form of play and communication. In Serbia, caregivers were aware of the importance of giving children personal attention and providing play, playthings, and communication. FGD participants stated that there were few resources for parents to help them provide these important things. These conditions were important to designing and implementing the parenting programs. However, changes were not made to the dosage or curriculum on the basis of this information.

### 3.2. Demographic Characteristics of Parents and Children Included in Outcomes Evaluation

Table 4 presents the demographic characteristics of participating caregivers and children before and at scale-up. In all countries, caregiver participants were overwhelmingly the child’s mother. The mothers’ average age ranged from 26 years in Zambia to 31 years in Bhutan and Serbia. Mothers in Bhutan and Zambia had an average of 6–8 years of formal education, while the average in Serbia was 14 years. The socioeconomic status of caregivers was measured in Bhutan and Zambia through ownership of contextually relevant household assets (e.g., bicycle, refrigerator, etc.). Out of 20 assets, caregivers owned an average of 11 in Bhutan and 6 in Zambia. Across all countries, the sample of children included an equal number of boys and girls. The average child in the sample was 15 months old in Bhutan and Zambia. These differences between program and comparison samples are accounted for in regression analyses for each country.

For Serbia and Zambia, a comparison of demographic characteristics by treatment status is presented in the Appendix A.

Not all households had participated in the parenting program though all were eligible. A question about program participation in the caregiver interview revealed that in Bhutan only 44% had attended at least one group session and only 30% had attended four or more sessions (see Table 5). In Serbia, 60% had received a home visit but only 20% of those surveyed received messages on child development, play and communication. In Zambia, coverage in program districts was estimated at 31% of eligible households for home visits, and 44% for attendance to a group session. This less-than-full participation required secondary regressions to be conducted on families from eligible sites who participated versus those who did not participate, despite being eligible.

### 3.3. Parent and Child Outcomes at Scale

#### 3.3.1. Bhutan Outcome Analyses

Table 6 presents regression results where the dependent variable is the HOME score at endline for a panel of households, controlling for a series of demographic and socioeconomic characteristics and a district fixed effect (control variable coefficients not shown, please refer to Appendix A for full output). At scale, all districts received the program, and we do not have a comparison group. Consequently, the HOME scores of those attending at least one session were compared with those who attended no session. As the table indicates, participation in at least one parenting session is associated with an increase of 0.54 points on the HOME inventory. This is equivalent to approximately 0.13 standard deviations. If caregivers attended at least 4 or more sessions (around 30% of the sample), they were likely to have seen an increase of 0.62 points on the HOME inventory, or around 0.15 of a standard deviation.

Table 7 presents the marginal effects drawing from the regression analysis, showing estimated mean outcome levels between caregivers who participated in at least one parenting session, as compared to those who did not participate at all (regression Models 1 and 3), as well as estimated mean outcomes for caregivers at different levels of dosage of the program (Models 2 and 4).

Child outcome results as measured by GSED and expressed in DAZ scores (models 3 and 4 in Table 6 showed small effects for caregivers who had attended at least one session vs. those who attended none, and for those who attended 4 or more sessions vs. those who attended none. As the table indicates, attendance of at least one session was associated with a 0.19 higher DAZ score at endline and an effect size of d = 0.19. This effect persisted at higher levels of attendance (4 or more sessions), indicating that, as compared to no attendance, there was a similar difference in outcomes associated with program participation, regardless of the number of sessions attended.

##### Sensitivity Checks for Bhutan

Our sensitivity checks examine whether the probability of being a program participant and attending more sessions are determined by any observed demographic characteristics, such as the caregiver’s education, age, child gender or their socio-economic status, as measured by the wealth asset score. As Table 8 shows, program participants were less wealthy, on average, than non-participants. While this estimated difference is minimal, it may have a somewhat dampening effect on the impact of the program, as higher socioeconomic status is associated with higher parenting outcomes and child developmental outcomes, in general. The program participant group was also more likely to have female children; however, child gender had not been shown to be predictive of child developmental outcomes on the GSED.

#### 3.3.2. Serbia Outcome Analyses

In Serbia, the Playful Parenting program was rolled out to a set of 34 municipalities across the country, based on a series of criteria that included the local government’s demonstrated interest and commitment to parenting programming, as well as the resources available to sustain it beyond the initial program implementation period. The sample for program sites was drawn from eight program municipalities who scored just above the cutoff for inclusion, and a comparison sample was drawn from four municipalities just below the cutoff score for inclusion in the program. Further, because the nurse visits were received by around 60% of the targeted eligible parents (sample of children <18 months), and only about 20% reported hearing a message about play and communication, we examined the effect on these subgroups.

As Table 9 demonstrates, there was virtually no difference in outcomes between program and comparison site eligible families at endline, when relevant characteristics were controlled (Model 1). Considering only the program municipalities, we were likewise unable to register any differences associated with having received a nurse’s visit (Model 2). However, Model 3 shows an adjusted mean difference of 1.25 points on the HOME score higher for those caregivers who reported having received messaging on play and responsive stimulation from their home visiting nurse. This should be interpreted with caution due the small subgroup sample size (n = 46). Regressions on child outcomes as measured through GSED at endline showed no differences on the development for age z-scores between subgroups, neither on the intention to treat (Model 4), nor subgroup models (Models 5 and 6).

Table 10 shows the means on key outcome measures by subgroup in Serbia, including by intention to treat analysis and by participation status. 

##### Sensitivity Checks for Serbia

For Serbia’s program participation, we examine the likelihood that the provider’s visit was determined by some of the family demographics (see Table 11). Indeed, we found that disability, as reported by the caregiver, substantially increased the likelihood of a nurse visit having taken place. This is not unexpected: the nurses, as general health practitioners, were likely paying closer attention to families with children with disabilities. Families who received a visit were less likely to have been Roma or another ethnicity; however, ethnicity was not a predictor of the likelihood of having received a visit where the caregiver recalled receiving counseling on play and responsive stimulation—which was associated with higher parenting outcomes.

#### 3.3.3. Zambia Outcome Analyses

In the Zambia program, scale up took place within the two selected districts (Katete and Petauke). The comparison sites were two waitlisted districts. As Table 12 indicates, the Intention to Treat analysis (Model 1) at scale showed an adjusted mean difference of 1.25 points on the HOME inventory between caregivers residing in the program target areas compared to those residing in comparison sites (confounding variables such as observed demographic characteristics are included as controls). This translated to an estimated 25% of a standard deviation effect size. Secondary analyses were conducted on participants within treatment sites who participated or did not in the program. Only 36% of the at-scale sample of eligible families reported having received a home visit in the previous year, and 44% had participated in at least 1 group session at either a health facility or in their community. Within treatment sites, no notable differences were found to be associated with participation in home visits. However, a nearly 3-point difference was estimated between caregivers who attended group sessions (*p* < 0.01), as compared to caregivers who received no program intervention, translating to an effect size of 0.62. While both modalities were offered by the program in the target districts, caregivers had to opt in to the group sessions at health facilities or community centers, while the home visits by volunteer frontline providers were determined independently of the household, by the frontline provider responsible for their catchment area.

Table 13 shows the subgroup means and standard deviations on key outcomes in Zambia. As the table indicates, and as the regression results suggest, outcomes on the HOME are higher by about 3 points within program areas for caregivers who participated in a group session. 

No corresponding differences were registered in child outcomes, as measured on the GSED using the Development for Age z-score (DAZ) (Table 12). Child outcomes appeared to be indistinguishable between families residing in program or comparison areas and not related to caregiver participation in the program.

##### Sensitivity Checks for Zambia

When we examine the probability of receiving a home visit or attending a parenting group session as a function of demographic characteristics (Table 14) none of the observed variables emerge as strong predictors of participation. Each additional month of child age increased the likelihood of participation by an average of 1%. Socioeconomic status, as measured by the mothers’ education and the household wealth index, was not predictive of program participation.

In summary, intention-to-treat analyses showed a small difference in parenting practices measured by the HOME Inventory in Zambia, but no other differences in outcomes. Because many eligible families reported that they had not participated in the program sessions (or received home visits), secondary analyses compared those in program sites who did and did not participate. These analyses revealed that the children in Bhutanese families who participated had a small difference in their GSED scores associated with the program. In Serbia, Serbian mothers who received messages on play had a small associated difference in their practices, and Zambian caregivers who reported having attended group sessions had a moderate difference in their parenting practices compared to peers who had not participated. Sensitivity checks examined differences between participants and non-participants on all observable characteristics, and did not find evidence of selection bias.

### 3.4. Caregivers Exposure and Learning at Scale

To better understand what caregivers learned during parenting sessions that they did attend or receive, we asked follow-up questions in caregiver surveys on whether they learned anything new, and if so, what they learned. Further, toward the end of the programs in mid-2024, we conducted FGDs in each country to explore caregivers’ exposure to the program and their reported learning. Although respondents to the FGDs and surveys were not always program participants, our analysis here is restricted to those who participated in the program. Their perspectives provide a glimpse into their experience with the programs.

#### 3.4.1. Bhutan Caregiver Learning

In Bhutan’s focus group discussions, parents recalled messages on play and responsive stimulation: one mother emphasized the importance of “free movement and play” for children’s physical and mental development, while another highlighted the value of consistent communication, explaining that “constant communication is key”. Talking to children frequently aids in their language development from as early as a few months old. Positive discipline also emerged in caregiver accounts, with one father sharing, “earlier, I used to scold my child when they would not listen, but now I talk calmly and explain things.”

#### 3.4.2. Serbia Caregiver Learning

In Serbia, by contrast, most caregivers who responded to the caregiver survey did not recall learning new content during their interactions with home-visiting nurses. Among those who did, few reported new knowledge gained about play and responsive stimulation. Instead, responses typically focused on other topics, such as nutrition or environmental concerns. However, some respondents did recall playful parenting messages linked to the program, such as a mother who said, “She [the nurse] emphasized that children should be communicated with from day one and that they should be talked to, that children react to conversation.” A FGD mother recalled being told: *“if you talk, if the sisters are involved, if the husband is involved, if we play, what we play with,”* and urged to *“don’t just be quiet. Whatever you do, talk to the baby.”*

#### 3.4.3. Zambia Caregiver Learning 

In Zambia, only about one third of caregivers reported learning something new during home visits by their CBVs, with roughly half of these responses related to playful parenting practices. In contrast, nearly all of the 44% of caregivers who attended a group session during the same period—roughly twice as many—reported learning something new, with a similar proportion (roughly half) mentioning an example of learning focused on play or early communication, e.g., “[I learned that I should] always talk to [the] child and teach new things.” FGDs further highlighted learning around motor skill development, with one mother explaining, “for a child to develop well mentally, as a mother, we teach the child many things, like playing ball, teaching the child running, and games using hands.”

In summary, these quotes help illustrate how the program modalities manifested differently among target caregivers. Caregivers who attended group parenting sessions in Bhutan and Zambia were more likely to report having learned something new about parenting, compared to caregivers who received a home visit in Serbia and Zambia. Because caregivers generally exercise greater agency over whether to participate in group sessions, compared to receiving a scheduled home visit, it is possible that their participation is driven in part by an interest in learning new parenting content—which in turn may also influence their recollection of what they learned. Still, the fact that only a small proportion of respondents in Serbia and Zambia recalled learning anything about play and early responsive stimulation during home visits carries important implications for future program uptake and reach in these countries.

## 4. Discussion

Across the three programs introducing playful parenting to caregivers of children birth to 3 years of age, effects were largely non-significant in intention-to-treat analyses. The one exception was in Zambia where there was a small effect on the HOME Inventory measure of parental practices. Because the programs did not reach a large number of eligible families, a secondary analysis was conducted on those who said they had participated in comparison to those who had not. In these analyses, small effects on the HOME Inventory were found in Bhutan and Serbia, and a moderate effect on those with group attendance in Zambia. Overall, the program yielded no impacts on child development in Serbia and Zambia and a small effect in Bhutan. Generally, these findings are disappointing and considerably lower than have been reported in meta-analyses [4,9]. However, recall that scaled programs generally report small effect sizes of 0.10 or less [5,18,19,20,21]. Explanations for these unexpectedly small impacts often rest on the design of the program (curriculum, dosage), low quality delivery by providers, and a workforce that is inadequately trained and supervised [18,21]. Added to these common explanations is the dilution due to expanding scale. Although there was evidence of effectiveness in the pilot evaluation in Bhutan, suggesting that scaling might have diluted impact, the programs in Serbia and Zambia had not been evaluated prior to scale. So, the program design itself might have contributed to low impact. Possible explanations for the findings are now presented. Three explanatory issues are worth discussing further. One is that the low uptake of programs implemented at scale often resulted in less than 60% of eligible families receiving the parenting program. A second issue is the need for pilot evaluations which help to understand whether the design of the curriculum and dosage are effective with this population. The third issue is how workforce issues might explain low impacts as a result of poor delivery quality and other implementation processes.

### 4.1. Coverage of Programs

The sample sizes estimated for data collection were based on expected coverage of programs and expected effect sizes. However, two conditions reduced our expectations of reach: one was that effects at scale cannot be expected to attain levels found in meta-analyses of effectiveness studies [4]; and the second was that programs at scale often do not reach eligible families [30]. The first concerns expectations of effect at scale. Although child development effect sizes found in meta-analyses of parenting programs are on average *d* = 0.40 in LMIC, those interventions are more controlled than the currently studied scaled programs—controlled in the sense of providing more training and supervision of the workforce and demanding more adherence among families. None of these scaled programs had supportive supervision, and quality was variable [6]. The second point concerns lack of coverage, namely the extent to which the program reaches its intended target population. Up to half of eligible families, selected randomly from treated sites had not actually participated in the program. It is recognized that maintaining high attendance at scale can be a challenge [20,30] and training sufficient numbers of providers is one of the greatest barriers to horizontal scale. Low attendance was the main challenge in Bhutan, whereas insufficient numbers of providers was the main barrier in Serbia and Zambia. Ways to improve coverage resulting from provider shortage (as in Zambia and Serbia) therefore include strategies to expand and improve training of providers, maintaining delivery quality through supportive supervision, and retaining workers with incentives. Ways to address low attendance (as in Bhutan) may include systematic enrollment of families after the birth of a child, community mobilization through advocacy, and support from opinion leaders.

### 4.2. Design Problems

The second issue is the need for pilot evaluations which help to understand whether the design of the curriculum and dosage are effective with this population. Bhutan’s pilot evaluation had indicated that their 12-session structured curriculum and dosage yielded changes in parenting practices and child development. Although the measures used in that study were not strong, they did suggest that the curriculum was suited to the population of Bhutan. A structured curriculum delivered over 12 sessions is considered appropriate for LMIC interventions [16]. In contrast, neither Serbia nor Zambia programs had conducted a pilot evaluation of the current program. Yet, a meta-analysis of the Care for Child Development curriculum used in these two countries found it to have inconsistent effects, largely non-significant due to inadequate dosage, and a curriculum that is unsuited to families who do not already provide responsive stimulation at home [25]. According to our FGDs before scale-up, Serbian parents did understand the benefits of play but not of being responsive, whereas Zambian parents tended to provide less play at home. The Serbian program did enhance the message about responsiveness in their program, so the curriculum might have been appropriate, but the dosage was a problem because it entailed five visits in the newborn period when responsive play was more limited than later in infancy. Zambian parents and providers initially knew little about responsive play and so required a more structured curriculum with higher dosage in order to have an impact. These limitations could have been identified in formative research, comparable to our FGD, and in an early pilot evaluation and remedied before scale-up. In Zambia, where formative research shows low levels of responsive stimulation at home, decisions about curriculum and dosage could have been made initially and after a pilot evaluation. A structured curriculum with many specific play and communication activities would be helpful for caregivers in building a repertoire of stimulating things to do with their children. Behavior change techniques, such as demonstrations and coaching, give parents the opportunity to learn as they practice with their child. Showing games that use home-available materials as playthings helps mitigate the belief that store-bought toys are needed. Future interventions do not need to create their own manuals from scratch; many effective manuals exist online and can be found with the help of technical support.

### 4.3. Implementation Process Challenges

Explanations for low impact often focus on inadequate implementation of an otherwise effective program. The quality of implementation service delivery rests largely with the frontline providers [6]. It refers not only to the *fidelity* of implementation—because fidelity to a poor curriculum is unhelpful—but the skillful use of techniques that change caregiver practices, such as coaching parents as they engage in new play or talk with their child. The quality of service delivery was found to improve at scale in Bhutan and Serbia, where health professionals delivered the program, but remained overall moderately good in Zambia, where it was delivered by volunteers [7]. Still, of the three country samples in this study, only the Bhutanese caregivers were able to recall in large numbers the play messages they had heard. For those in Serbia, newborn messages about hygiene and breastfeeding may have diluted the play messages. Zambian providers were not trained to use behavior change techniques such as demonstration and coaching parents as they engaged in new play with their child. Thus, the messages in Zambia may not have been memorable to caregivers. Although this explanation is speculative, it points to workforce implementation processes related to training, monitoring and supervision that have been found to influence change among parents [16]. In order to transfer changes found in parents to their children’s development, parents must practice the new stimulating activities with their child on a regular basis. Requiring what is often called “homework” was implemented in Bhutan to good effect when homework was emphasized in the following session: parents were asked to show what new games they had played with their child since the last contact. This clearly did not happen in Serbia or Zambia where some effects on parental practices did not transfer to child development outcomes.

### 4.4. Strengths

One key contribution of this study is that it examined outcomes of programs that were delivered at scale. Very few parenting programs have been evaluated at scale [5], and many had small effect sizes of 0.10 or less [18,19,20,21]. By scale, we mean that programs in this study had met their goal after four years to expand horizontally across districts/municipalities and to expand vertically through the health system from workforce to government. Some publications have examined their implementation process as they transitioned to scale [7].

Second, we examined outcomes across three different programs using conventional measures such as the HOME Inventory to measure parenting practices and the GSED to measure child development, along with large sample sizes. The use of standardized, internationally validated measures contributes to the global knowledge base on programmatic approaches to strengthen positive parenting practices.

### 4.5. Limitations

There are several important limitations to this study. First, the study was structured around the scale up processes selected by each program implementer. Consequently, sites and families were not randomly assigned to treatment and comparison conditions, rendering it not possible to set up an experimental framework, and limiting options for comparison groups. Bhutan, for example, delivered the program to all districts nation-wide at scale, so a comparison group was not feasible. In Serbia and Zambia, comparison sites were not intentionally receiving a playful parenting program, though they might have received other programs such as newborn health in Serbia and nutrition in Zambia. Consequently, lack of random assignment introduced potential biases that could explain some of the results, and the lack of control over comparison sites introduced potential contamination in these sites.

Another limitation was the high levels of non-participation among eligible families in treated sites, which lowered the possibility of finding impact with an intention-to-treat analysis. Consequently, in addition to an intention-to-treat analysis, we conducted a sub-analysis on those residing in treatment sites. Further, in all three settings, “treated” status was based on the caregivers’ report of participation in the program. In some cases, this might mean participation only once. Non-participation is common in programs that go to scale and therefore lack the control of efficacy studies [30]. Our sensitivity checks examining probability of participation did not show significant selection bias; the program participants tended to be slightly lower on the socioeconomic scale in Bhutan and Serbia, and more likely to have included families of children with disabilities in Serbia—all of which may have had a slightly dampening effect on outcomes. However, unobserved factors may have influenced who participated and who recalled the messages received, and this influence cannot be fully accounted for.

## 5. Conclusions and Recommendations for Practice and Research

The results of this study show that achieving impact on caregiver and child outcomes at scale continues to be a challenge. Findings show small or no impact on parents and children. Features related to the design of programs e.g., (dosage, curriculum) and implementation processes (e.g., workforce training, retaining, supervision and quality delivery) were potentially influential. It is important to ask whether ineffective programs are worth scaling. As an independent research and learning group, we did not make this decision; we evaluated the programs as implemented by partners. Below we offer some recommendations for future policy and programs and for research.

### 5.1. Recommendations for Policy and Programs

Several key recommendations emerge from this study on features to be prioritized in scaled parenting interventions. Recommendations focus on ensuring significant benefits for parents and children. First, the study clearly showed the importance of strong advocacy to build demand, given that need does not always imply demand. This is necessary to ensure caregiver enrollment and retention. Personal invitations to caregivers after the birth of their child ensure that they are aware of the program. Community mobilization with the help of opinion leaders, especially male leaders if father engagement is desired. Small incentives may be necessary along with an engaging program where caregivers quickly perceive new learnings and benefits.

A second recommendation is to ensure alignment of the design (curriculum, modality, dosage) with both caregivers and providers. Formative qualitative research is needed to reveal their current understanding of child development and responsive stimulation. A light-touch program and short provider training may be sufficient for contexts where knowledge is moderate among parents and providers. However, where such knowledge is not present, a more intense, yet scalable, dosage and structured curriculum is needed to promote new learning and practice among parents.

The third recommendation concerns the workforce. Sufficient numbers of providers need to be trained, retained and supervised to maintain a quality delivery for all families who are eligible. Training and supervision must be more intense and hands-on for volunteer providers than for professionals. However, all providers need to be assessed after training to ensure their competence in the content and the form of delivery.

The final recommendation is for all programs seeking impact at scale to conduct a pilot evaluation of the scalable version of the program within the same context, ascertain effectiveness, and then monitor indicators of parenting practices and child development outcomes on an annual basis. Ideally, a full evaluation of outcomes should be done by an independent entity to supplement internal program monitoring.

### 5.2. Recommendations for Research

This study offers an agenda for future implementation research on the effectiveness of parenting programs going to scale. More research is needed on aspects of programs that enhance effectiveness and facilitate scale up. Including fathers in parenting programs is an issue that many experts recognize as important but a severe challenge. In addition, the use of digital technology and other innovations to train providers and to deliver messages to caregivers should be studied. Finally, more research on top-down and bottom-up approaches to scale is needed: top-down programs start at the government level but often overlook effectiveness with caregivers, whereas bottom-up approaches seek to ensure effects with caregivers but struggle to achieve government adoption. A combination of these two approaches, grounded in system and country context, may facilitate scale up with impact on outcomes.

## Figures and Tables

**Table 1:** Dosage and duration of playful parenting programs in the study.

Country and Program	Coverage at Scale	Intervention Intensity	Proportion of Play in Session Content
Bhutan Prescription to Play, Save the Children	Nationwide (20 districts)	12 monthly group sessions at health facility run by health professionals	9 sessions lasting ~25–60 min each
Serbia Playful Parenting, UNICEF Serbia	34 municipalities (out of 145)	5 home visits by nurses in first 15 days 1 visit in 1st year 1 visit in 2nd year	As determined by visiting nurse
Zambia Care for Child Health Growth and Development	2 districts within Eastern Province	7 home visits by community-based volunteers; or 4 group sessions at health facility (25–40 min)	Follows Care for Child Development (CCD) curriculum; illustrations and questions about play in each session

**Table 2:** Frequency of references to provisions supporting child development in caregivers FGDs before scale up.

Provisions	Bhutan	Serbia	Zambia
Food	26	8	24
Health and hygiene	20	2	10
Play (playthings)	39 (19)	49 (18)	18 (17)
Talk with child	35	29	10
Love	3	16	0
Give attention	1	23	0
Social life with peers	19	5	1
Motor activities	3	10	3

**Table 3:** Frequency of references to resources needed and available in caregivers FGDs before scale up.

Resources/Supports Needed by Caregivers	Bhutan	Serbia	Zambia
Food, playthings	3	0	19
Caregiver training	16	9	2
Outside childcare	16	3	6
**Resources/supports available to parents**			
Health system	4	19	2
Information apps	0	3	0

**Table 4:** Caregiver and child demographic characteristics prior to and at scale.

	Bhutan	Serbia	Zambia
**Pre-scale characteristics**			
Respondent is child’s mother (%)	97.0	83.1	93.0
Mother: Age	30.7	31.1	25.9
Mother: Education (years)	7.5	13.6	5.9
Wealth: Asset index (max. 20)	10.5	-	5.9
Child is a girl (%)	50	45.3	50
Child age in months	14.8	11.7	14.9
N	432	698	1010
**At-scale characteristics**			
Respondent is child’s mother (%)	96.3	94.2	99.5
Mother: Age	32.0	30.2	26.4
Mother: Education (years)	7.5	12.5	6.4
Wealth: Asset index (max. 20)	11.1	-	6.2
Wealth: Can afford extra things	-	73.7	-
Child is a girl (%)	50	51.9	48
Child age in months	28.2	11.6	18.8
N	432	636	1024

**Table 5:** Program participation.

Type of Participation	Bhutan (n = 432)	Serbia (n = 205)	Zambia (n = 638)
Percent of caregivers reached by provider (at least one session or one home visit)	44% had attended at least one group session	63% (any nurse visit)	31% (provider home visit in past 12 months)
	30% had attended four or more sessions	23% (nurse visit with play content)	44% (attended a group session in past 12 months)

**Table 6:** Bhutan. Regression coefficients for caregiver outcomes (HOME Inventory) and child development outcomes (GSED DAZ) at scale based on program participation.

Variables Entered (Partial List)	HOME Score: Any Program Participation in Past 12 Months	HOME Score: Dosage of Program Sessions (Reference Category Is No Participation)	GSED DAZ: Any Program Participation in Past 12 Months	GSED DAZ: Dosage of Program Sessions (Reference Category Is No Participation)
Attended at least 1 group session	0.54 ** (0.18) *d* = 0.13 (0.04, 0.21)		0.18 ** (0.06) *d* = 0.18 (0.06, 0.30)	
1–3 sessions		0.38 (0.57)		0.23 * (0.10) *d* = 0.23 (0.03, 0.43)
4 or more sessions		0.62 ** (0.21) *d* = 0.15 (0.05, 0.25)		0.16 ** (0.05) *d* = 0.16 (0.06, 0.26)
Constant	34.71 *** (0.14)	34.71 *** (0.14)	0.75 *** (0.03)	0.75 *** (0.03)
N	432	432	432	432

Note: * *p* < 0.10; ** *p* < 0.05; *** *p* < 0.01. Standard errors clustered at the district level in parentheses. Models control for child covariates (sex and age) and caregiver covariates, including mother’s age and educational attainment, and household socioeconomic status (measured by the number of locally relevant assets). All models include district fixed effects. For statistically significant coefficients, Cohen’s *d* is calculated to express the coefficient as an effect size. A 95% confidence interval for the effect size is included in parentheses below *d*.

**Table 7:** Bhutan. Estimated subgroup means (SD) and sample sizes for subgroups by program participation status.

	(1)	(2)	(3)	(4)
Outcome Variables	Did Not Participate	Attended at Least One Session	Attended 1–3 Sessions	Attended 4+ Sessions
HOME score (out of 45)	35.0 (2.1)	35.6 (2.1)	35.3 (2.2)	35.7 (2.2)
GSED for Age Z-score (DAZ)	0.77 (0.21)	0.96 (0.21)	0.98 (0.20)	0.95 (0.20)
N	231	201	71	130

**Table 8:** Sensitivity checks for program participation in Bhutan.

Demographic Variables	Prob. of Attending a Group Session	Number of Sessions
Mother’s age	−0.00 (0.00)	−0.06 (0.03)
Mother’s years of education	−0.00 (0.00)	−0.08 (0.05)
Father’s age	0.00 (0.00)	0.01 (0.03)
Father’s years of education	0.00 (0.00)	0.02 (0.05)
Assets Score	−0.03 ** (0.01)	−0.17 ** (0.06)
Child is a girl	0.05 (0.03)	0.36 (0.46)
Child’s age in months	−0.01 (0.00)	−0.02 (0.04)
Constant	0.48 *** (0.02)	2.77 *** (0.21)
Observations	432	432

Note: ** *p* < 0.05; *** *p* < 0.01. Standard errors clustered at the district level in parentheses.

**Table 9:** Serbia. Estimated regression coefficients for caregiver outcomes (HOME inventory) and child outcomes (GSED) at scale based on program participation.

	(1)	(2)	(3)	(4)	(5)	(6)
Variables Entered	HOME: ITT	HOME: TOT/Nursing Visit	HOME: TOT/Nursing Visit with Play	DAZ: ITT	DAZ: TOT/Nursing Visit	DAZ: TOT/Nursing Visit with Play
Program	0.10 (0.34) d = 0.02 (−0.12, 0.16)			−0.01 (0.09) d = −0.01 (−0.17, 0.15)		
Nurse visit		−0.54 (0.54) d = −0.12 (−0.36, 0.12)			0.04 (0.18) d = 0.03 (−0.30, 0.36)	
Play visit			1.25 * (0.69) d = 0.29 (−0.30, 0.60)			0.04 (0.18) d = 0.04 (−0.14, 0.22)
Constant	37.15 *** (0.73)	33.10 *** (2.16)	32.72 *** (2.03)	0.33 ** (0.16)	0.30 * (0.47)	0.30 (0.46)
N	614	198	198	614	198	198

Note: * *p* < 0.10; ** *p* < 0.05; *** *p* < 0.01. Standard errors (SE) in parentheses. Models control for child covariates (sex and age) and caregiver covariates, mother’s age and educational attainment, and household socioeconomic status (measured by the number of locally relevant assets). Continuous covariates are centered at their sample means to aid interpretation of the intercept. The sample in Models 2, 3, 5, and 6 is restricted to caregivers residing in program-receiving municipalities. For statistically significant coefficients, Cohen’s d is calculated to express the coefficient as an effect size. A 95% confidence interval for the effect size is included in parentheses below *d*.

**Table 10:** Serbia. Estimated subgroup means (SD) and sample sizes for subgroups by program participation status.

	By Intention to Treat Status		By Participation Status (Within Program Areas)		
Outcome Variables	Program Municipality	Comparison Municipality	Did Not Receive Visit	Any Nurse Visit	Nurse Visit with Play Content
HOME score (out of 45)	38.6 (2.8)	38.5 (2.8)	39.1 (2.7)	38.6 (2.7)	39.7 (2.9)
GSED for Age Z-score (DAZ)	0.33 (0.38)	0.34 (0.38)	0.31 (0.59)	0.35 (0.59)	0.37 (0.59)
N	246	390	74	124	46

**Table 11:** Serbia sensitivity check: Probability of program participation based on demographic variables.

Demographic Variables	Nursing Visit	Nursing Visit with Play
Child’s age	−0.07 *** (0.01)	−0.03 ***(0.01)
Child is girl	0.03 (0.06)	0.04 (0.06)
Disability	0.52 *** (0.08)	0.92 *** (0.09)
Mother’s age	−0.01 (0.00)	−0.00 (0.01)
Mother’s years of education	0.00 (0.01)	0.01 (0.01)
Less well off	0.08 (0.07)	−0.05 (0.08)
Roma or Other (non-Serbian)	−0.30 ** (0.13)	0.09 (0.15)
Constant	0.33 ** (0.14)	0.32 ** (0.15)
N	198	198

Note: ** *p* < 0.05; *** *p* < 0.01. Regression coefficient; standard errors in parentheses.

**Table 12:** Zambia. Regression coefficients (SE) for caregiver outcomes (HOME inventory) and child outcomes (GSED) at scale based on program participation.

	(1)	(2)	(3)	(4)
Variables Entered	HOME Score ITT	HOME Score TOT	GSED DAZ ITT	GSED DAZ TOT
Program district	1.25 *** (0.31) d = 0.25 (0.13, 0.37)		0.05 (0.06)	
Visited by a CBV (past 12 mo.)		0.66 * (0.39) d = 0.13 (−0.02, 0.28)		−0.10 (0.09)
Attended at least one group session (past 12 mo.)		2.96 *** (0.37) d = 0.62 (0.47, 0.77)		0.08 (0.08)
Constant	25.50 *** (0.29)	25.34 *** (0.37)	−0.41 *** (0.06)	−0.30 *** (0.08)
N	1022	636	1022	636

Note: * *p* < 0.10; *** *p* < 0.01. Standard errors (SE) in parentheses. Models control for child covariates (sex and age) and caregiver covariates, mother’s age and educational attainment, and household socioeconomic status (measured by the number of locally relevant assets). The sample in TOT Models (2) and (4) is restricted to caregiver residing in program-receiving districts. For statistically significant coefficients, Cohen’s d is calculated to express the coefficient as an effect size. A 95% confidence interval for the effect size is included in parentheses below d.

**Table 13:** Zambia. Estimated subgroup means (SD) and sample sizes for subgroups by program participation status.

	By Intention to Treat Status		By Participation Status (Within Program Areas)		
Outcome Variables	Program Districts	Comparison Districts	Did Not Participate	Received Home Visit	Participated in Group Session
HOME score (out of 45)	26.7 (1.8)	25.5 (1.8)	25.2 (1.8)	27.1 (2.4)	28.4 (1.9)
GSED for Age Z-score (DAZ)	−0.32 (0.23)	−0.37 (0.23)	−0.34 (0.21)	−0.40 (0.22)	−0.29 (0.22)
N	638	386	288	201	255

**Table 14:** Zambia sensitivity checks: Probability of program participation as a function of demographic variables.

Demographic Variables	Probability of Receiving a CBV Home Visit	Probability of Attending a Group Session
Child’s age (in months)	0.01 *** (0.00)	−0.00 (0.00)
Child is a girl	−0.03 (0.04)	−0.01 (0.04)
Mother’s age	−0.00 (0.00)	0.00 (0.00)
Mother’s years of education	0.01 (0.01)	0.00 (0.01)
Assets score	0.01 (0.01)	0.01 (0.01)
Constant	0.38 *** (0.03)	0.47 *** (0.03)
N	636	636

Note: *** *p* < 0.01. Regression coefficients; standard errors in parentheses.

**Figure A1:**
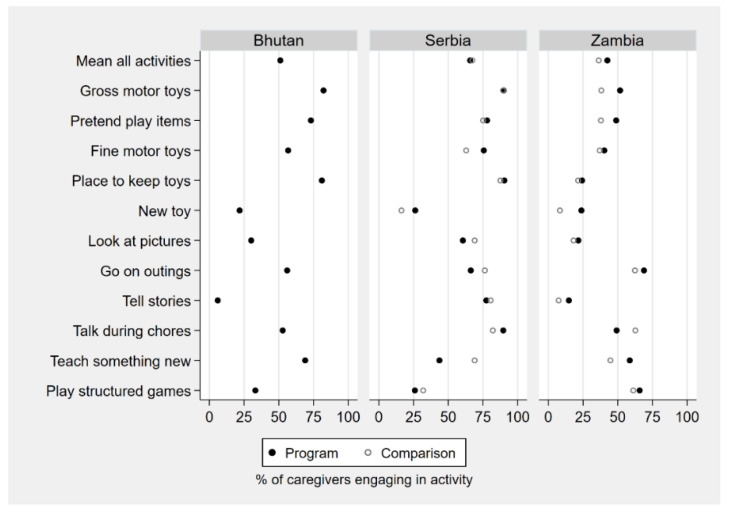
Caregiver engagement in stimulating activities by country before scale-up. Note: Serbia sample restricted to children under 18 months of age. No comparison group is available in Bhutan.

## Data Availability

Data may be available upon request to the corresponding author (comoeva@fhi360.org).

## Appendix A

**Table A1:** Caregiver outcomes by country before scale-up.

Outcome Variables	Bhutan	Serbia	Zambia
Total HOME score (out of 45)	30.4 (5.6)	-	27.2 (5.8)
Stimulation score (out of 15)	-	11.2 (3.3)	-
Score on common items (out of 11)	5.6 (2.3)	7.3 (2.3)	4.5 (2.5)
N	432	698	1010

**Table A2:** Caregiver outcomes by program status before scale-up.

	Bhutan		Serbia		Zambia	
Outcome Variables	Phase 1 Pilot	Phase 2–3 Scale	Program	Comparison	Program	Comparison
Total HOME score (out of 45)	32.0 *** (5.0)	29.3 (5.3)			27.9 *** (5.9)	25.7 (5.4)
Stimulation score (out of 15)			11.1 (3.4)	11.2 (3.2)		
Score on common items (out of 11)	6.1 *** (2.2)	5.3 (2.3)	7.2 (2.4)	7.4 (2.3)	4.7 *** (2.6)	4.0 (2.3)
N	179	253	385	313	675	335

Note: *** *p* < 0.01. Stars indicate statistically significant differences in means between program and comparison areas within each country, except in Bhutan, where the comparison is between Phase 1 and Phases 2–3. Standard deviation (SD) in parentheses.

**Table A3:** Serbia. Demographic characteristics by treatment status before scale-up.

Demographic Variables	Program	Comparison	Difference
**Caregiver characteristics**			
Mother’s Age	30.9	31.3	0.3
Mother’s Education (years)	13.9	13.2	−0.7 ***
Welfare (%)	68.9%	48.3%	−0.2 ***
Ethnically Serbian	90.9%	94.6%	0.0 *
**Child characteristics**			
Child is a girl (%)	44.9%	45.7%	0.0
Child's age in months	11.9	11.4	−0.5 *
Child has disability	0.3%	1.0%	0.0
N	385	313	

Note: * *p* < 0.05; *** *p* < 0.001.

**Table A4:** Zambia. Demographic characteristics at baseline by treatment status before scale-up.

	Program	Comparison	Difference (C-P)
**Caregiver characteristics**			
Respondent is child’s mother (%)	0.93	0.94	0.01
Mother’s Age	26.05	25.51	−0.54
Mother’s Education (years)	5.33	7.10	1.77 ***
Father’s Age	32.84	32.31	−0.53
Father’s Education (years)	6.11	8.11	2.00 ***
Wealth: Asset index (max. 20)	5.74	6.10	0.36
**Child characteristics**			
Child is a girl (%)	0.52	0.47	−0.05
Child's age in months	15.14	14.58	−0.56
N	675	335	1010

Note: *** *p* < 0.01.

**Table A5:** Serbia. Demographic characteristics at endline by treatment status at scale.

Demographic Variables	Program	Comparison	Difference (C-P)
**Caregiver characteristics**			
Mother’s Age	30.5	30.0	−0.5
Mother’s Education (years)	11.9	12.9	1.0 ***
Wealth: Poorer	20.3%	29.2%	0.1 **
Wealth: Higher	39.7%	36.3%	0.0
Wealth: Highest	38.4%	30.0%	−0.1 **
Ethnically Serbian	91.9%	89.2%	0.0
**Child characteristics**			
Child is a girl (%)	0.5	0.5	0.0
Child's age in months	11.9	11.5	−0.5 *
Child has disability	0.0	0.0	0.0
N	246	390	

Note: * *p* < 0.10; ** *p* < 0.05; *** *p* < 0.01.

**Table A6:** Zambia. Demographic characteristics at endline by treatment status at scale.

	Program	Comparison	Difference (C-P)
**Caregiver characteristics**			
Respondent is child’s mother (%)	0.99	1.00	0.00
Mother’s Age	26.50	26.16	−0.34
Mother’s Education (years)	5.86	7.39	1.53 ***
Father’s Age	32.32	32.67	0.34
Father’s Education (years)	6.42	8.80	2.38 ***
Wealth: Asset index (max. 20)	6.10	6.34	0.24
**Child characteristics**			
Child is a girl (%)	0.47	0.49	0.02
Child's age in months	19.63 ***	17.55	−2.08 ***
N	638	386	

Note: *** *p* < 0.01.
